# Lycopene Alleviates Depression‐Like Behavior in Chronic Social Defeat Stress‐Induced Mice by Promoting Synaptic Plasticity via the BDNF–TrkB Pathway

**DOI:** 10.1002/fsn3.70003

**Published:** 2025-01-22

**Authors:** Heyan Xu, Yuna Wang, Dandan Geng, Fengming Chen, Yujia Chen, Lisa Cynthia Niwenahisemo, Lei Shi, Ning Du, Ziqiang He, Xiaoming Xu, Li Kuang

**Affiliations:** ^1^ Psychiatric Center The First Affiliated Hospital of Chongqing Medical University Chongqing China; ^2^ Department of Psychiatry The First Affiliated Hospital of Chongqing Medical University Chongqing China; ^3^ Medical Sciences Research Center University‐Town Hospital of Chongqing Medical University Chongqing China; ^4^ Hubei University of Traditional Chinese Medicine Affiliated Shiyan Hospital Shiyan China; ^5^ School of Communications and Information Engineering Chongqing University of Posts and Telecommunications Chongqing China

**Keywords:** depression, lycopene, synaptic plasticity

## Abstract

Lycopene is a natural plant extract widely studied for its powerful antioxidant and neuroprotective effects. Emerging evidence suggests that it also possesses potential antidepressant properties. Compared to commonly used clinical antidepressants, lycopene offers higher safety; however, its underlying mechanisms remain unclear. Therefore, this study aims to explore the mechanisms through which lycopene exerts its antidepressant effects. We employed the chronic social defeat stress (CSDS) model to induce depressive‐like behaviors in mice, followed by lycopene treatment (20 mg/kg). Based on previous research, we focused on synaptic plasticity by examining the expression of synaptic proteins in the hippocampus to uncover potential mechanisms. The results showed that CSDS induced synaptic plasticity impairments in the hippocampus but lycopene treatment significantly improved these synaptic deficits and reversed the depressive‐like behaviors induced by CSDS. Moreover, lycopene treatment upregulated the expression of brain‐derived neurotrophic factor (BDNF) and reduced the activity of BDNF–TrkB/pTrkB pathway in the hippocampus. These molecular changes were consistent with changes in synaptic‐related proteins, suggesting that lycopene may enhance synaptic plasticity via the BDNF–TrkB/pTrkB signaling pathway. This study explored the mechanisms underlying depressive‐like behaviors induced by CSDS in mice and provided preclinical evidence that lycopene may serve as a potential antidepressant. It offers an effective avenue for the development of novel antidepressant therapies.

## Graphical abstract

This study demonstrated lycopene could alleviate depression‐like behavior in chronic social defeat stress‐induced mice by promoting synaptic plasticity via the BDNF–TrkB pathway.

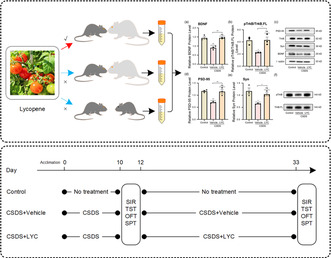

**Abbreviations**
BDNF: brain‐derived neurotrophic factor
CSDS: chronic social defeat stress
LPS: lipopolysaccharide
PSD‐95: post‐synaptic density protein 95
pTrkB: phosphorylated tropomyosin receptor kinase B
PVDF: polyvinylidene Fluoride
qRT‐PCR: quantitative reverse transcription polymerase chain reaction
RIPA: radioimmunoprecipitation assay
Syn: synaptophysin
TBST: tris‐buffered saline with Tween 20
TrkB: tropomyosin receptor kinase B

## 1 Introduction

Depression is a mental disorder characterized by low mood, slowed thinking, and reduced willpower, often accompanied by symptoms such as fatigue, loss of appetite, and insomnia (James et al. 2018). In China, the lifetime prevalence of depression is 6.8%, and the annual prevalence is 3.6%, making it one of the leading causes of loss of healthy life years (Nagy et al. 2020). The World Health Organization predicts that by 2030, depression will become the leading cause of disease burden worldwide (Malhi and Mann 2018). However, the exact pathogenesis of depression remains unclear. The monoamine hypothesis suggests that depression is related to a deficiency of neurotransmitters in the brain, such as serotonin, norepinephrine, and dopamine, which has led to the development of antidepressant drugs. However, these drugs have limited efficacy and significant side effects (Warden et al. 2007). In addition, genetic factors, psychosocial factors, the hypothalamic–pituitary–adrenal (HPA) axis, and oxidative stress also play essential roles in the onset and progression of depression (Cui et al. 2024). Among them, psychosocial factors are most common in human life, such as unemployment, divorce, and the death of loved ones, which can act as chronic stressors and trigger depression (Cui et al. 2024; Slavich and Sacher 2019). Although the mechanisms linking psychosocial factors and depression are not yet fully understood, studies have shown that depression is associated with neural network dysfunction and a reduction in synapse numbers (Wang, Chen, et al. 2023). Furthermore, some antidepressants, such as imipramine and fluoxetine, can reverse stress‐induced synapse loss, suggesting that preventing structural and functional changes in neurons caused by stress may be vital in treating depression (Fries et al. 2023; McEwen, Nasca, and Gray 2016; Moda‐Sava et al. 2019). Previous research has shown a close relationship between depression and synaptic plasticity (Duman et al. 2016; Ru et al. 2022; Yan et al. 2021). Synaptic plasticity refers to specific changes in synaptic structure and function in response to neural activity, one of the brain's most important functions (Duman et al. 2016). Impaired synaptic plasticity can lead to depressive symptoms, such as anhedonia, cognitive impairment, and behavioral despair (Lim et al. 2012; Yuen et al. 2012). Therefore, protecting or restoring synaptic plasticity may be a new strategy for treating depression.

Currently, the primary clinical treatment for depression remains pharmacological therapy, with most drugs based on the monoamine hypothesis. These drugs often have slow onset, limited efficacy, and numerous side effects, leading to a growing interest in finding new therapeutic options (Warden et al. 2007). Influenced by the traditional Chinese medicine concept of “medicine and food homology,” researchers have focused on natural plant extracts. Compared to synthetic drugs, natural plant extracts offer the advantages of being suitable for long‐term consumption, having fewer side effects, and being safer. As research has progressed, plant extracts such as lycopene and curcumin have been proven to have neuroprotective effects (Fan et al. 2019; Lin et al. 2014; Samarghandian et al. 2017), with lycopene standing out due to its powerful antioxidant properties and wide availability. Lycopene is a natural pigment widely found in plants such as tomatoes and watermelons. It is insoluble in water but soluble in lipids. Animal experiments have shown that orally administered lycopene enters the digestive tract of mice, where it is primarily absorbed in the intestine and converted into chylomicrons that enter systemic circulation. It is mainly distributed in the liver, plasma, kidneys, lungs, and brain (Ford, Elsen, and Erdman 2013). Guo et al. (2019) administered lycopene orally at a dose of 8 mg/kg to mice and found that its plasma concentration peaked at 6 h post‐administration, with a half‐life of 8.29 h. Furthermore, lycopene, with its strong anti‐inflammatory and antioxidant properties, can cross the blood–brain barrier, offering significant potential in cardiovascular diseases, cancer, and neurological disorders (Chen, Huang, and Chen 2019; Lin et al. 2018; Saini et al. 2020). Recent studies have shown that lycopene can alleviate LPS‐induced depressive‐like symptoms in mice during the tail suspension test and forced swim test (Zhang et al. 2016), and clinical studies have demonstrated an inverse correlation between carotenoid intake and depressive symptoms (Yu et al. 2022), suggesting that lycopene is a potential antidepressant. Compared to conventional antidepressants, lycopene has the advantages of low toxicity and high safety, although its specific mechanism of action remains unclear. Zhao et al. (2018) found that oral supplementation with lycopene could reverse neuronal damage and synaptic dysfunction in aged mice, while Guo et al. (2023) demonstrated that brain‐targeted administration of lycopene could inhibit amyloid‐β production and restore synaptic plasticity. These studies indicate that lycopene holds great potential in improving synaptic plasticity. Given the close relationship between synaptic plasticity and depression, we hypothesize that lycopene may exert its antidepressant effects by improving synaptic plasticity. To test this hypothesis, we designed the present study. In contrast to the previous study carried out by Zhang et al. (2016), we employed the chronic social defeat stress (CSDS) model and oral administration to explore the antidepressant mechanism of lycopene, aiming to understand this compound's effects better and lay the foundation for its future clinical application.

## 2 Materials and Methods

### 2.1 Animals

Aggressive CD‐1 mice and C57BL/6J were purchased from Beijing Vital River Laboratory Animal Technology Co. Ltd. Both were male. The C57BL/6J mice were 6–8 weeks old, weighing 20–24 g, and the CD‐1 mice were 4–6 months old, weighing 25–29 g. All animals were housed in temperature‐controlled (22°C ± 2°C) animal rooms with automatic ventilation systems, at five mice per cage. Lights in the animal rooms were on from 8:00 AM to 8:00 PM, and mice were given ample food and water. All experiments were conducted after a 7‐day acclimation period. All experimental procedures involving animals were approved by the Institutional Animal Care and Use Committee (IACUC) of Chongqing Medical University (Approval No. IACUC‐CQMU‐2023‐09025) and were conducted in strict accordance with the National Institutes of Health Guide for the Care and Use of Laboratory Animals.

### 2.2 CSDS Procedure

Three days were spent on screening aggressive CD‐1 mice before starting the formal experiment. Seven‐week‐old male C57BL/6J mice (not used in the main experiment) were placed into the cages of the CD‐1 mice to be screened. In the next 5 min, the latency, intensity, and frequency of attacks by the CD‐1 mice were observed and recorded. This screening was repeated three times with a one‐day interval between each round. CD‐1 mice with moderate attack intensity, short latency, and high frequency of attacks were selected and placed individually into specially designed cages for acclimatization. After acclimatization, C57 mice were randomly assigned to either a control group (*n* = 24) or a model group (*n* = 60). The model group was placed in a specially designed cage with CD‐1 mice for 5–10 min of fighting, with the duration adjusted based on the aggression level of the CD‐1 mice. Mice in the control group were randomly paired (1:1 ratio) and placed in the same cage type for 5–10 min of interaction. After the fighting, the CD‐1 mice and the C57 mice were separated by a transparent acrylic board with holes, allowing them to see and smell each other, and were kept in this setup for 24 h. The following day, before the next fighting session, the C57 mice were exchanged with those in neighboring cages to prevent adaptation between the C57 and CD‐1 mice. The control group was treated similarly, with mice separated by perforated transparent boards and exchanged daily. This procedure was repeated for 10 consecutive days. Fresh food and drinking water were provided regularly during the modeling period, ensuring stable environmental factors. When the depression‐like model in the mice was fully established, the model group was randomly split into the CSDS model group (CSDS + Vehicle) and the lycopene administration group (CSDS + LYC).

### 2.3 Preparation of Drug

Lycopene was purchased from Shanghai Yuanye Bio‐Technology Co. Ltd. (B20378). Lycopene was dissolved in corn oil for later use. After the CSDS modeling was completed, the CSDS + LYC group was given lycopene (20 mg/kg) daily by oral gavage, while the CSDS + Vehicle group received an equivalent volume of solvent (Corn oil). The control group was not given any drug or solvent administration.

### 2.4 Behavioral Tests

#### 2.4.1 Sucrose Preference Test (SPT)

The sucrose preference test is a classic method used to assess anhedonia in mice (Liu et al. 2018). Prior to the formal experiment, a 2‐day acclimation period is conducted. 1% sucrose is dissolved in water for use, named sugar water. In the initial 24 h, mice are offered two bottles of sugar water for acclimation. In the next 24 h, one sugar water is substituted with pure water. Following acclimation, all C57BL/6J mice are subjected to 24 h of fasting and water deprivation to induce hunger and thirst.

During the formal experiment, each mouse was given one bottle of pure water, another bottle of sugar water, and fresh food. Mice were allowed to drink and eat freely while maintaining stable environmental conditions for 24 h. The bottles were weighed before and after the experiment, and then the intake values of pure and sugar water could be obtained by calculating the change in bottle weight. The sucrose preference was calculated using the formula: sucrose preference = (sucrose intake/(sucrose intake + water intake)) × 100%.

#### 2.4.2 Open‐Field Test (OFT)

The OFT is employed to assess locomotor activity, exploratory behavior, and anxiety levels in mice within an unfamiliar environment (Horka et al. 2024). The open field apparatus consists of a white, unroofed box measuring 50 × 50 × 50 cm, with the bottom divided into 25 squares of 5 × 5 cm each. During the test, the mouse was gently placed into the open field and allowed to move freely for 6 min. The first 2 min were used for adaptation and we recorded the last 4 min. The total distance traveled by the mouse was calculated by Smart 3.0 software. After each mouse, the bottom and walls of the box were wiped with 75% ethanol to prevent odor residues from affecting the activity trajectory of subsequent mice.

#### 2.4.3 Tail Suspension Test (TST)

The TST is used to assess despair behavior in mice, with more severe depression showing more pronounced signs of despair. In the test, the mouse's tail was taped at the posterior 1/3 and suspended from support against a white background, with the head of the mouse positioned 30 cm above the platform. The mouse was suspended for 6 min, and the total immobile time during the last 4 min was recorded.

#### 2.4.4 Social Interaction Test (SIT)

The SIT is primarily used to evaluate the social behavior of mice induced by CSDS. Normal C57BL/6J mice typically exhibit a series of exploratory behaviors when faced with a novel environment, and CD‐1 mice in the test will show curiosity and frequent contact. However, after repeated social defeat, C57BL/6J mice will significantly reduce their exploration of novel environments and display noticeable fear towards CD‐1 mice, demonstrating social avoidance.

##### 2.4.4.1 Test Design

Two white boxes measuring 50 × 50 × 50 cm with open tops were prepared. In one box, a transparent perforated box containing CD‐1 mice was placed in the center of one side of the bottom, and the surrounding squares were defined as the interaction area. A transparent empty box with the exact specifications was placed symmetrically in the other box. At the start of the test, a C57BL/6J mouse was gently placed next to the empty box and the time it spent in the interaction area (No Target, T1) was observed and recorded for 150 s. Afterward, the mouse was removed and returned to its home cage for a 30‐s rest, and the box was wiped with 75% ethanol to prevent affecting the next mouse. The rested C57BL/6J mouse was then placed in the box with CD‐1 mice for 150 s, and the total duration of time spent in the interaction zone (Target, T2) was recorded. The social interaction ratio (SIR) was calculated as follows:

SIR=T2/T1

### 2.5 Nissl Staining

The brain tissues of C57BL/6J mice were embedded in paraffin and sectioned into 4 μm thick slices. After dewaxing and rehydration, the tissue sections were immersed in a staining solution for 2–5 min, washed with water, differentiated with 0.1% acetic acid, and then washed with tap water to stop the reaction. The sections were then dried and cleared in xylene for 10 min and mounted with neutral resin. Under the microscope (Leica DM2000), the Nissl bodies and neuronal changes in the hippocampal regions were observed, and images were captured at 20× magnification using an optical microscope. The cell count was quantified using ImageJ analysis software (ImageJ 1.54f).

### 2.6 Western Blotting (WB)

All mice were sacrificed after the final behavioral test to collect brain tissues. All brain tissues were lysed on ice using RIPA lysis buffer (Beyotime, Shanghai, China) to extract proteins, and protein concentrations were then measured using a BCA protein assay kit (Beyotime). Protein samples were resolved using SDS‐polyacrylamide gel electrophoresis (Sangon, Shanghai, China) and then transferred onto PVDF membranes (Millipore, Burlington, MA, USA) and then incubated with 5% non‐fat milk (Solarbio, Beijing, China) to block non‐specific binding and then exposed to a range of primary antibodies overnight at 4°C. The primary antibodies list: anti‐Syn (1:20,000; Proteintech, Wuhan, Hubei, China), anti‐PSD‐95 (1:10,000; Proteintech), anti‐BDNF (1:3000; Huabio, Hangzhou, Zhejiang, China), anti‐TrkB (1:1000; Huabio), anti‐pTrkB (1:2000; Huabio), and anti‐tubulin (1:10,000; Abcam, Cambridge, UK). Following washing with TBST (5 times, 5 min each), the membranes were incubated at room temperature with a secondary antibody (1:6000, goat anti‐rabbit; Boster, Wuhan, Hubei, China) for 1 h, and protein bands were visualized using enhanced chemiluminescence (ECL). Band intensities were measured using ImageJ analysis software.

### 2.7 Quantitative Real‐Time Polymerase Chain Reaction Analyses (qRT‐PCR)

Total RNA was extracted from the tissues using the Trizol method (Invitrogen, USA). Following the manufacturer's guidelines, total RNA was converted into cDNA using the PrimeScript RT reagent Kit (TaKaRa, Japan). Quantitative mRNA expression was carried out with a SYBR Green PCR kit (TaKaRa) and measured using the SYBR Green detection system (Roche, Germany). All samples were run in triplicate. The results were normalized against β‐actin, and relative gene expression was determined using the $2^{-\Delta \Delta C_{t}}$ method. The mouse primers specific to each gene are detailed in Table 1.

**TABLE 1:** The primer sequences.

Gene	Forward	Reverse
*BDNF*	ACGACGACATCACTGGCTGAC	AGGCTCCAAAGGCACTTGACTG
*TrkB.FL*	GGTGGCTGTGAAGACGCTGAAG	AATGTGCTCGTGCTGGAGGTTG
*PSD‐95*	AGAGGTAGCAGAGCAGGGGAAG	GGACGGATGAAGATGGCGATAGG
*Syn*	TGCCGCCAGACAGGAAACAC	CCAGAGCACCAGGTTCAGGAAG

### 2.8 Statistical Analysis

Data analysis was conducted with GraphPad Prism 8 software. All data are presented as mean ± standard error of the mean (SEM). The Shapiro–Wilk test was used to assess the normality of the data distribution. If *p* < 0.05, a non‐parametric test was employed. The Brown‐Forsythe test was used to evaluate the homogeneity of variances. When variances were homogeneous, one‐way ANOVA was carried out, and Tukey's post hoc test was applied afterward. If variances were not homogeneous, the Welch's ANOVA was applied, and further comparisons were made using Dunnett's T3 multiple comparison test. *p* < 0.05 was determined to be significantly different.

## 3 Results and Discussion

### 3.1 CSDS‐Induced Depression‐Like Behavior in Mice

**FIGURE 1:**
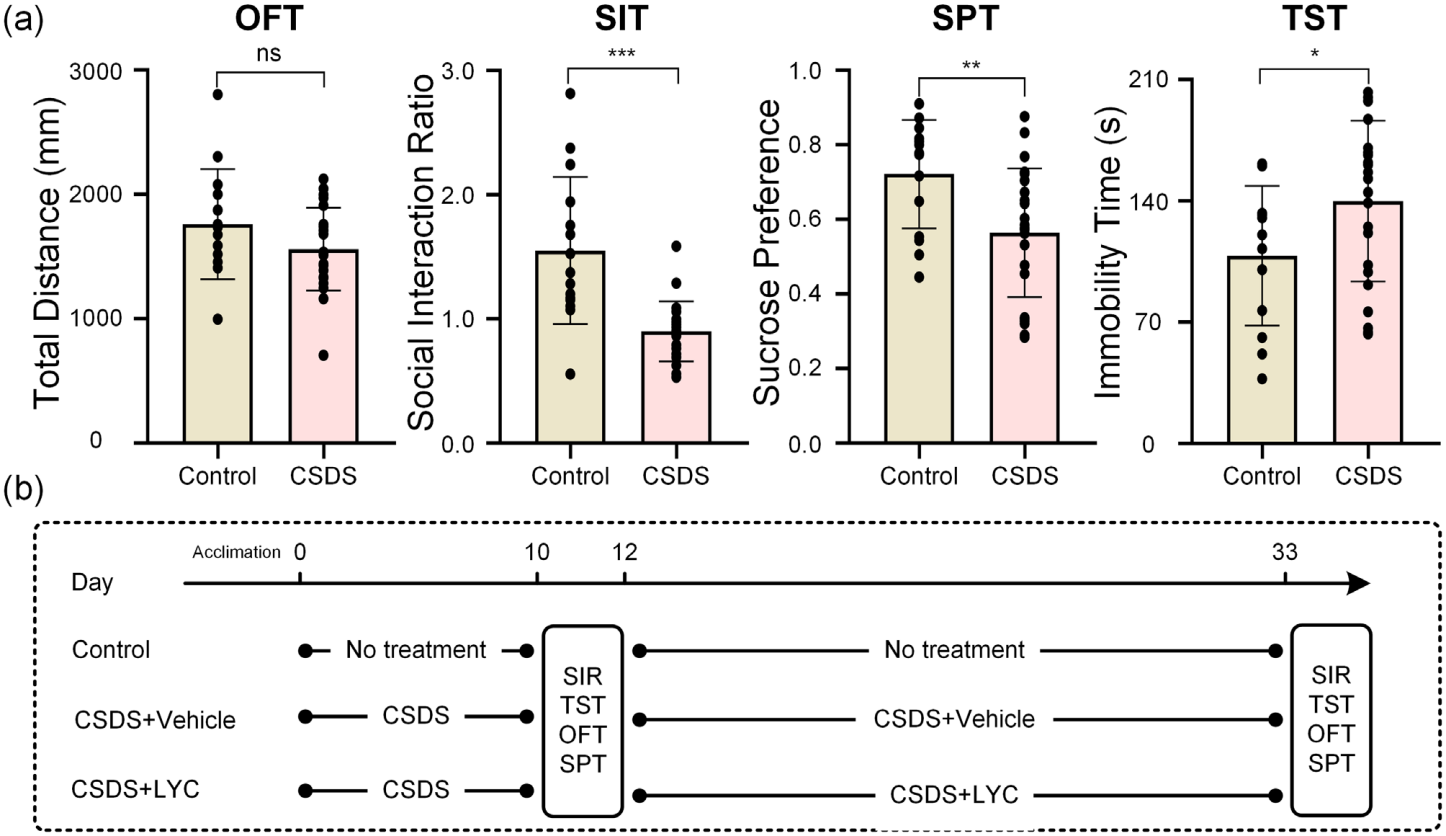
Behavioral tests in CSDS‐induced mice and research procedure. OFT, open field test; SIT, social interaction test; SPT, sucrose preference test; TST, tail suspension test. (a) Results of behavioral tests, the total distance in the OFT, the social interaction ratio in the SIT, the sucrose preference in the SPT and the immobility time in the TST. (b) Schematic representation of the CSDS procedure and treatments in mice. All the data are expressed as mean ± SEM (*n* = 24 in the control group *n* = 60 in the CSDS group). *p < 0.05, **p < 0.01, ***p < 0.001, ns means non‐significant versus control group.

Behavioral tests were conducted immediately after 10 days of CSDS modeling, with results shown in Figure 1a. In the SIT, the SIR was significantly decreased in mice subjected to CSDS (*t*_38_ = 4.878, *p* < 0.001), indicating that these mice spent less time in the chamber with the CD‐1 mouse (T2) compared to controls. Under normal conditions, driven by their social instincts, animals are more inclined to explore social stimuli (Horka et al. 2024), repeatedly staying in the area where the CD‐1 mouse is located. However, mice exposed to CSDS displayed social avoidance, avoiding the unfamiliar CD‐1 mouse, reflecting a core symptom of human depression—social withdrawal. In contrast, the control group mice had relatively higher SIRs, exhibiting normal exploratory behavior in response to social stimuli.

Similarly, the SPT and the TST, respectively, reflect anhedonia and hopelessness, both of which are the core symptoms of depression. In the SPT (*t*_38_ = 2.953, *p* = 0.005), mice subjected to CSDS demonstrated a reduced preference for sucrose relative to the control group, while in the TST (*t*_38_ = 2.046, *p* = 0.048), they exhibited longer immobility times. The OFT is primarily used to assess anxiety‐like behavior in mice. Although anxiety was not the main focus of this study, the overall distance traveled by the mice in the open field can indirectly reflect their activity levels. Our results showed no statistically significant differences between the two groups of mice in the OFT (*t*_38_ = 1.583, *p* = 0.122).

### 3.2 Effects of Lycopene on Behavioral Performance of Stressed Mice

**FIGURE 2:**
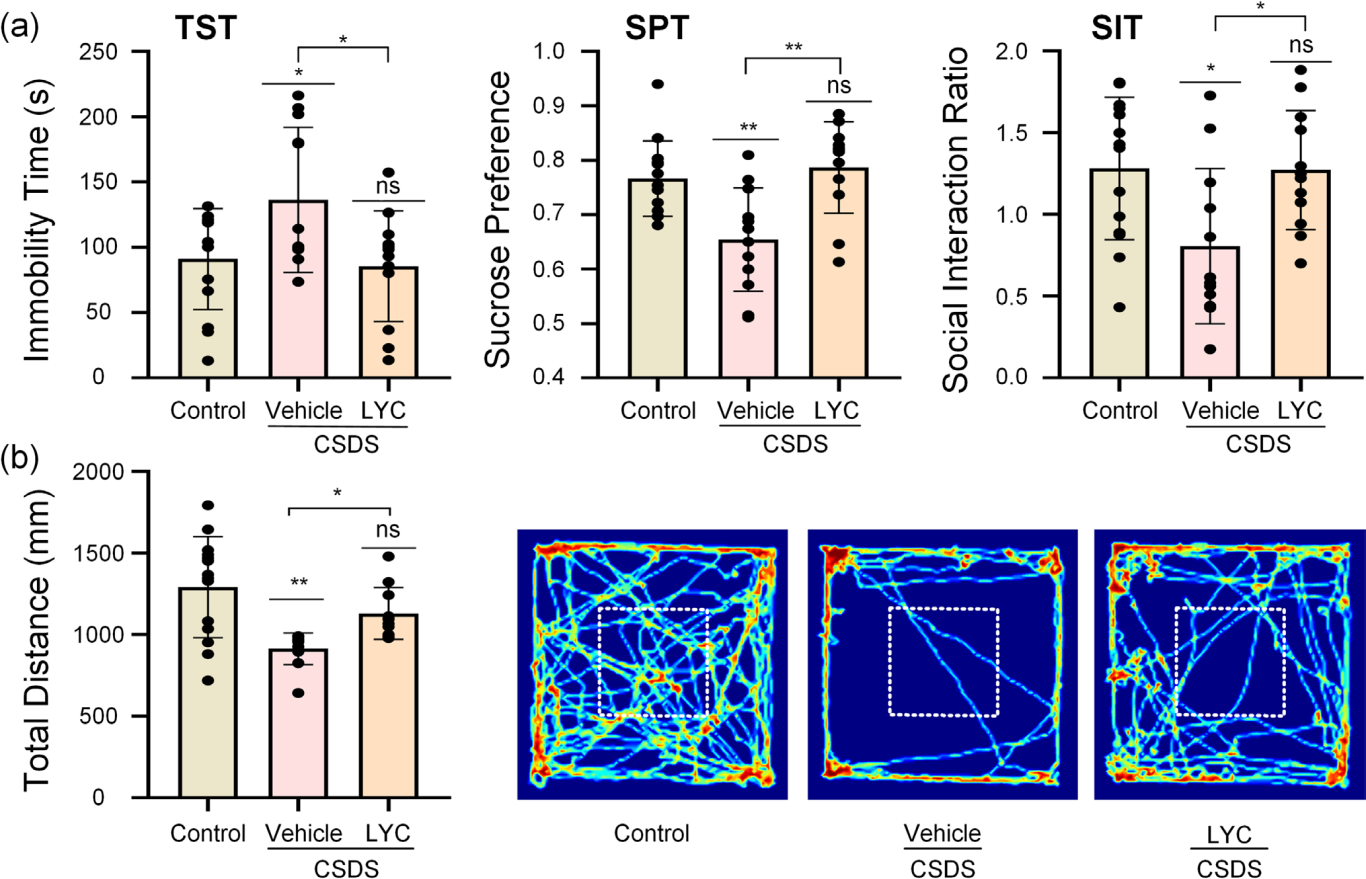
Effects of lycopene on CSDS‐induced depressive behaviors. OFT, open field test; SIT, social interaction test; SPT, sucrose preference test; TST, tail suspension test. (a) The immobility time in the TST, the sucrose preference in the SPT, and the social interaction ratio in the SIT. (b) The total distance in the OFT and heat maps of path tracing in the OFT after lycopene administration. All the data are expressed as mean ± SEM (*n* = 12 per group). *p < 0.05, **p < 0.01, ns means non‐significant versus control group, or CSDS + Vehicle versus CSDS + LYC.

To investigate the effects of lycopene on depression, we conducted a series of behavioral tests following the end of drug treatment again. As shown in Figure 2, in the SIT (*F*_2,35_ = 4.984, *p* = 0.013), the SIR of the CSDS + vehicle group mice remained lower compared to the control group (*p* = 0.021). In contrast, the SIR of the CSDS + LYC group mice significantly increased (*p* = 0.03), indicating that lycopene can improve social avoidance behavior induced by CSDS. Similarly, in the SPT and the TST, the group treated with lycopene (CSDS + LYC) showed improved performance. In the SPT (*F*_2,35_ = 9.096, *p* < 0.001), sucrose consumption was markedly reduced in CSDS‐exposed mice (*p* = 0.004), but lycopene intervention considerably increased the sucrose preference in mice (*p* = 0.001). The TST involves placing mice in an environment they find aversive and cannot escape. Initially, mice from all groups attempt to escape. However, after a period of adaptation, they transition to a passive state, characterized by a natural hanging posture with minimal limb movement, reflecting a state of despair. We assessed the level of depression in mice by recording the total duration of this despair state. As expected, compared to the control group, the immobility time was significantly increased in the CSDS + vehicle group (*F*_2,35_ = 4.525, *p* = 0.018, *p* = 0.043), while lycopene administration reduced this increase (*p* = 0.027). In the OFT (*F*_2,35_ = 9853, *p* < 0.001), mice in the CSDS + vehicle group reduced the activity distance significantly compared to the control group (*p* = 0.003). Conversely, the distance traveled by mice treated with lycopene increased (*p* = 0.046).

### 3.3 Effects of Lycopene on the Histomorphology of the Hippocampus in Stressed Mice

**FIGURE 3:**
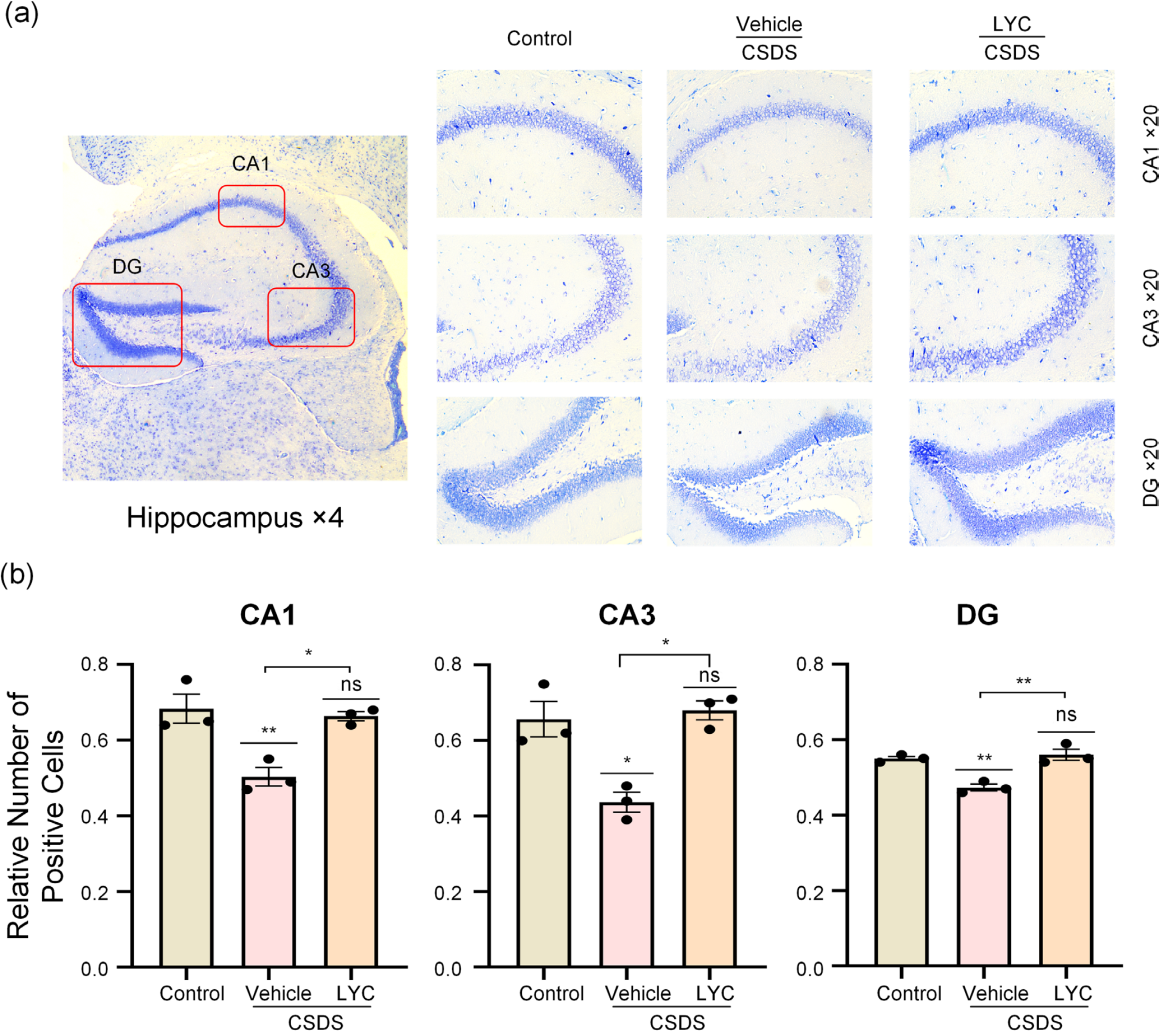
Neuroprotective effect of lycopene in different hippocampal subregions. (a) Nissl staining in the hippocampal CA1, CA3 and DG subregions. (b) The statistical analysis in CA1, CA3 and DG subregions. Data are presented as mean ± SEM (*n* = 3 per group). *p < 0.05, **p < 0.01, ns means non‐significant versus control group, or CSDS + Vehicle versus CSDS + LYC.

Nissl staining was used to observe the pathological morphological changes in the hippocampal tissue of mice. As depicted in Figure 3, the hippocampal neurons of the control group mice were numerous and tightly arranged, with intact cell morphology, clear cell structures, and abundant Nissl bodies. In contrast, the CSDS + Vehicle group mice exhibited sparse neuron arrangement, reduced neuron count (CA1: *F*_2,6_ = 13.27, *p* = 0.006, *p* = 0.008, CA3: *F*_2,6_ = 15.35, *p* = 0.004, *p* = 0.01, DG: *F*_2,6_ = 19.58, *p* = 0.002, *p* = 0.006), and disorganized or diminished Nissl bodies with unclear boundaries compared to the control group. Treatment with lycopene improved the pathological damage in the hippocampal regions induced by CSDS, with a significant increase in the number of positive cells (CA1: *p* = 0.014, CA3: *p* = 0.006, DG: *p* = 0.003), more organized neuron arrangement, more apparent Nissl body morphology, and an increased number of Nissl bodies.

### 3.4 Effects of Lycopene on Hippocampal Neuronal Synaptic Plasticity in Stressed Mice

**FIGURE 4:**
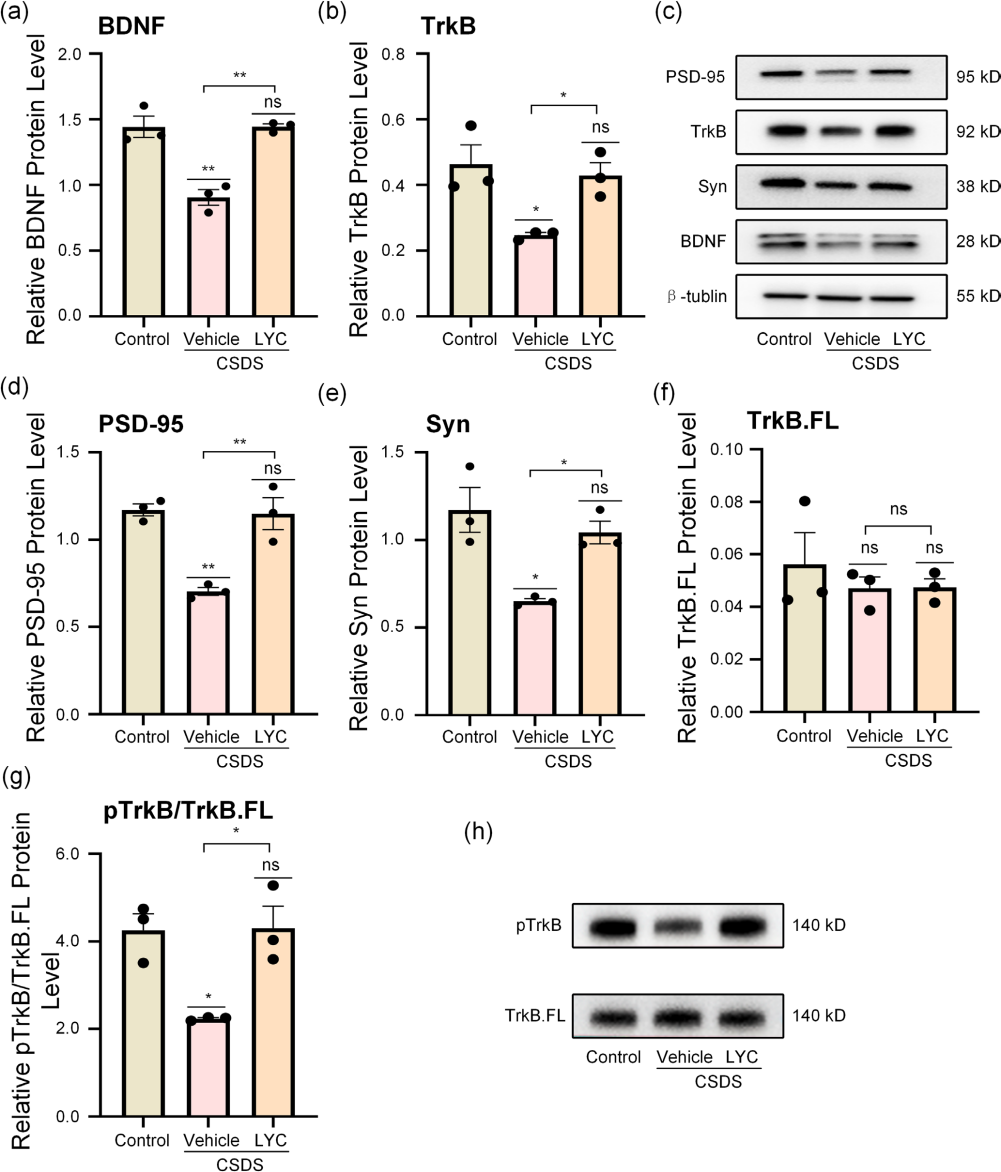
Effects of lycopene on synaptic proteins and BDNF–TrkB signaling pathway. (a, b, d–g) Protein level of BDNF, TrkB, PSD‐95, Syn, TrkB.FL, pTrkB/TrkB.FL. (c, h) Representative WB bands. Data are presented as mean ± SEM (*n* = 3 per group). *p < 0.05, **p < 0.01, ns means non‐significant versus control group, or CSDS + Vehicle versus CSDS + LYC.

WB was used to detect the hippocampal expression levels of synaptic proteins Syn and PSD‐95 in mice, reflecting lycopene's effects on hippocampal synaptic plasticity. As the results shown in Figure 4, the expression levels of synaptic proteins in the CSDS + Vehicle group mice were markedly downregulated compared to the control group (PSD‐95: *F*_2,6_ = 20.74, *p* = 0.002, *p* = 0.003, Syn: *F*_2,6_ = 10.57, *p* = 0.011, *p* = 0.011), demonstrating marked synaptic plasticity damage in the stress‐induced mice. In contrast, the hippocampal expression levels of synaptic proteins in the CSDS + LYC group were significantly upregulated compared to the CSDS + Vehicle group (PSD‐95: *p* = 0.004, Syn: *p* = 0.037), suggesting that lycopene treatment improved the synaptic plasticity damage in the hippocampus of mice exposed to CSDS.

**FIGURE 5:**
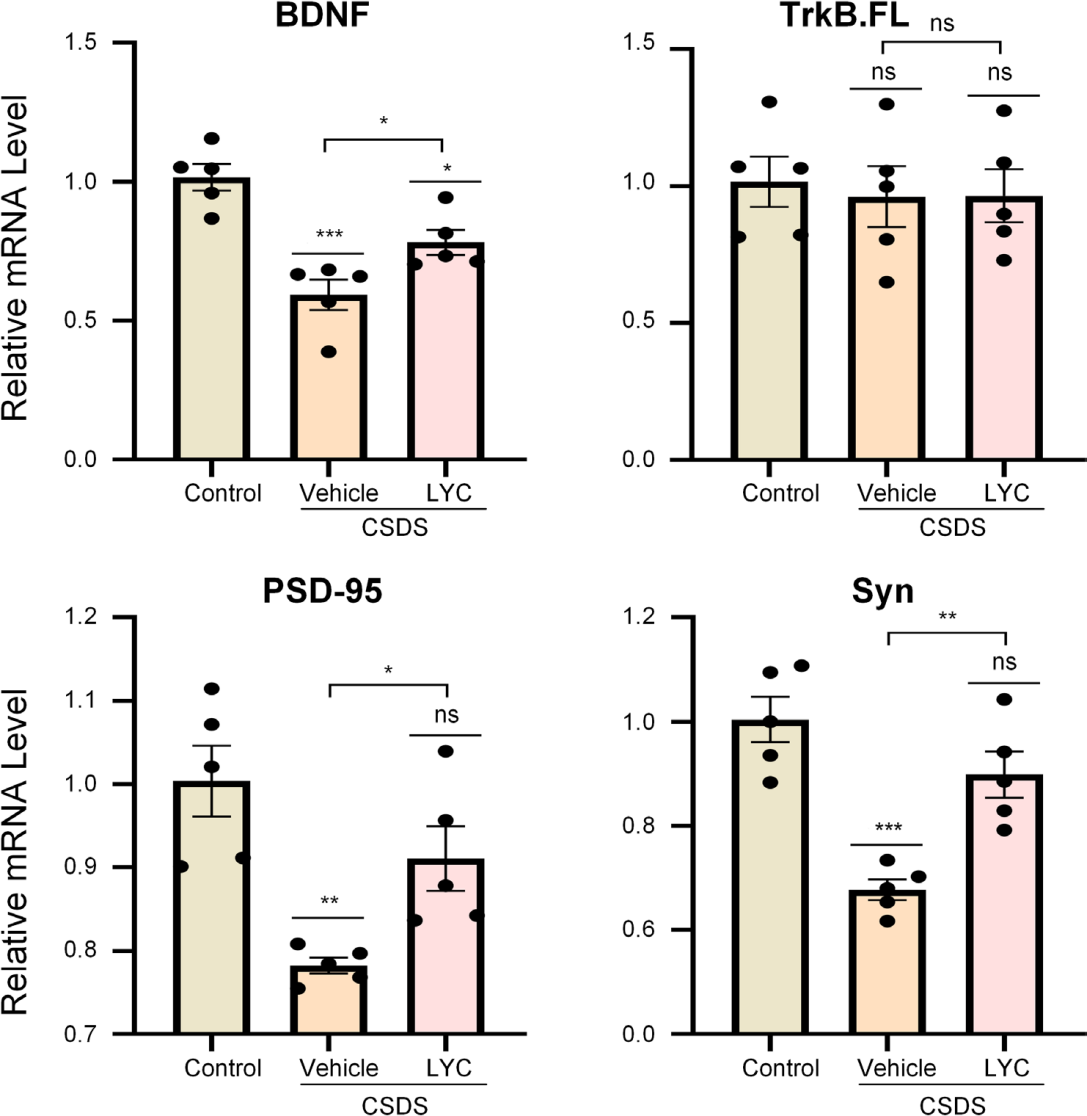
Effect of lycopene on mRNA in CSDS‐induced mice. Data are presented as mean ± SEM (*n* = 5 per group). *p < 0.05, **p < 0.01, ***p < 0.001, ns means non‐significant versus control group, or CSDS + Vehicle versus CSDS + LYC.

Similarly, qRT‐PCR results confirmed this conclusion (Figure 5). The hippocampal mRNA expression levels of *PSD‐95* and *Syn* in the CSDS + Vehicle group mice were significantly downregulated compared to the control group (*PSD‐95*: *F*_2,12_ = 10.89, *p* = 0.002, *p* = 0.0015, *Syn*: *F*_2,12_ = 19.55, *p* < 0.001, *p* < 0.001), while the mRNA expression levels of *PSD‐95* and *Syn* in the hippocampus of the CSDS + LYC group mice were substantially upregulated compared to the model group (*PSD‐95*: *p* = 0.048, *Syn*: *p* = 0.004).

### 3.5 The Role of the BDNF–TrkB Pathway in the Antidepressant Effects of Lycopene

Research has shown that using lycopene can upregulate BDNF expression in aged CD‐1 mice (Zhao et al. 2018). Given the close relationship between BDNF and synaptic plasticity, we hypothesized that the BDNF–TrkB pathway plays a crucial role in lycopene's improvement of synaptic plasticity. To verify this hypothesis, we examined the expression levels of BDNF, TrkB, and pTrkB. As shown in Figure 4a, the expression level of BDNF in the CSDS + Vehicle group was significantly lower than that in the normal group (BDNF: *F*_2,6_ = 27.33, *p* = 0.001, *p* = 0.002). In contrast, BDNF expression in the CSDS + LYC group treated with lycopene was upregulated compared to the depression model group (BDNF: *p* = 0.002), indicating that lycopene treatment promotes BDNF secretion. Studies (Cao et al. 2020; Dorsey et al. 2012; Renn, Leitch, and Dorsey 2009) have shown that TrkB exists in two variants: the full‐length TrkB (TrkB.FL) and the truncated TrkB (TrkB.T1), which have different molecular weights. Here, we analyzed both variants separately. As shown in Figure 4f, TrkB.FL exhibited no significant changes among the groups, whereas TrkB.T1 expression was reduced in the CSDS + Vehicle group (TrkB.T1: *F*_2,6_ = 7.89, *p* = 0.021, *p* = 0.024) and upregulated following lycopene treatment (*p* = 0.048). Furthermore, to assess functional activity changes in the BDNF–TrkB pathway, we measured pTrkB levels. As shown in Figure 4g, the relative expression of pTrkB in the CSDS + Vehicle group was significantly decreased compared to the normal group (pTrkB: *F*_2,6_ = 7.90, *p* = 0.021, *p* = 0.024), but it was elevated in the CSDS + LYC group (*p* = 0.048). These findings suggest that the functional activity of the BDNF–TrkB pathway is inhibited in depression model mice and that lycopene treatment alleviates this inhibition.

Similarly, qRT‐PCR results confirmed this conclusion. As shown in Figure 5, the mRNA expression levels of *BDNF* were downregulated in CSDS‐induced mice (*F*_2,12_ = 18.26, *p* < 0.001, *p* < 0.001). However, following lycopene treatment, the mRNA expression levels of *BDNF* were markedly elevated compared to the model group (*p* = 0.049). The mRNA levels of *TrkB.FL* did not change significantly among the groups, which is consistent with the WB results.

## 4 Discussion

This research aims to investigate the antidepressant mechanisms of lycopene using the classic CSDS animal model of depression. Findings demonstrated that the CSDS paradigm damages hippocampal synaptic plasticity in mice. Furthermore, lycopene treatment was shown to mitigate this synaptic plasticity impairment, thereby reversing the depressive‐like behaviors induced by CSDS. In the CSDS + Vehicle group, the hippocampal expression of BDNF and pTrkB was markedly downregulated, but following lycopene treatment, the expression of BDNF and pTrkB increased. This suggests that lycopene may improve CSDS‐induced synaptic plasticity damage through the BDNF–TrkB pathway, thereby reversing depressive‐like behaviors. Furthermore, the Nissl staining results also showed that the lycopene treatment group exhibited a more normal tissue structure., further supporting the neuroprotective effects of lycopene.

According to Lin et al. (2014), lycopene exerted anti‐neuroinflammatory effects by activating the AMP‐activated protein kinase‐α1/heme oxygenase‐1 pathway. Building on this, Zhang et al. (2016) used LPS to trigger depressive‐like behaviors in mice (Zhang et al. 2016). They suggested that alleviating LPS‐triggered depressive‐like behaviors by lycopene might be related to the regulation of HO‐1. However, the depressive‐like symptoms involved in these studies were artificially induced by the chemical LPS, resulting in acute and transient depressive‐like behaviors in animals. In contrast, depression is a chronic, persistent disorder, and chronic stress paradigms are more reliable and effective in modeling depressive‐like behaviors in rodents compared to acute stress paradigms (Antoniuk et al. 2019; Atrooz, Alkadhi, and Salim 2021; Iñiguez et al. 2016). Additionally, recent research has suggested that chronic stress can cause structural and functional changes in neurons (Fries et al. 2023; McEwen, Nasca, and Gray 2016), which helps explain the persistent symptoms of depression. Addressing this, Kumar et al. (2019) employed a chronic unpredictable stress (CUS) paradigm to naturally induce depressive‐like symptoms in mice, where lycopene similarly exhibited significant antidepressant effects. These findings suggest that lycopene holds great potential as an antidepressant, yet its precise mechanisms are not entirely understood. In our study, we employed the CSDS model, which, along with the CUS model, is widely used as a classic animal model of depression. Compared to CUS, CSDS offers higher ecological validity and more substantial reproducibility. Following the induction of depression, mice were administered lycopene, with dosage levels (20 mg/kg) based on the study by Kumar et al. (2019), which has already confirmed the antidepressant effect and safety of this dosage. According to the FDA's proposed human equivalent dose (HED) (U.S. Food and Drug Administration [FDA] 2002), this dosage converts to 1.62 mg/kg for humans. Although there are currently no clinical studies on the use of lycopene for treating depression in humans, based on the toxicological studies of lycopene (Trumbo 2005), this is considered a safe dosage for humans. Behavioral results following lycopene treatment revealed that the immobility time in the TST was significantly reduced in the CSDS + LYC group compared to the CSDS + Vehicle group. Additionally, lycopene significantly improved CSDS‐induced social avoidance, and the sucrose preference index increased markedly. These findings indicate that lycopene can reverse depressive‐like behavior induced by CSDS. Notably, when measuring the total distance traveled in the OFT at the end of the CSDS procedure, no significant difference was observed between the CSDS group and the control group. However, after treatment, the total distance traveled by the CSDS + Vehicle group was significantly reduced compared to the control group. This conclusion may suggest that extending the duration of social defeat beyond the traditional 10‐day paradigm might yield more pronounced behavioral phenotype differences (Lu et al. 2021).

Synaptic plasticity is one of the essential hypotheses of depression (Cui et al. 2024). Synaptic loss and dysfunction are believed to be associated with major depressive disorder (MDD), and changes in synaptic density are related to the severity of depression and network alterations (Howard et al. 2019). Zhao et al. (2018) discovered that lycopene could improve synaptic plasticity and cognitive function, providing a new avenue for exploring its mechanism of action. These conclusions suggest that lycopene may exert antidepressant effects by enhancing synaptic plasticity, which is the basis for our study. We observed that in mice exposed to CSDS, pre‐ and post‐synaptic components were reduced, consistent with previous research (Iñiguez et al. 2016). However, after lycopene treatment, the reduction in synaptic‐related proteins induced by CSDS was significantly improved, demonstrating the potential of lycopene in enhancing synaptic plasticity. However, further investigation into the detailed mechanisms of its action is still needed.

BDNF is an essential neural plasticity and development protein, mediating neuronal maturation and synapse formation. Conversely, the production and release of BDNF are also regulated by neuronal activity (Castrén and Hen 2013). Two different types of receptors generally mediate BDNF signaling: the p75 neurotrophin receptor (p75NTR) and the TrkB receptor (Podyma et al. 2021). However, to our knowledge, the synaptic effects of BDNF are almost exclusively mediated by TrkB (Castrén and Hen 2013; Sairanen et al. 2005). TrkB signaling promotes the survival of newly generated neurons in the dentate gyrus, enhances axon and dendrite growth, stabilizes synapses, and facilitates synaptic transmission (Khalifeh et al. 2020). In our study, we examined the expression levels and functional changes of BDNF–TrkB/pTrkB. The results indicated that CSDS led to a downregulation of BDNF expression in the hippocampus of mice, accompanied by a concurrent decrease in pTrkB levels. The level of pTrkB reflects the activity of the BDNF signaling pathway. Under normal circumstances, BDNF binds to the extracellular domain of the TrkB.FL receptor, inducing autophosphorylation of TrkB.FL, which exposes binding domains for PLCγ, adenosine triphosphate (ATP) and growth factor receptor‐bound protein 2 (GRB2) (Wang, Kavalali, and Monteggia 2023), thereby propagating the signal downstream. Thus, the reduction in pTrkB expression in depressive model mice may primarily result from the decreased BDNF levels. Following lycopene treatment, BDNF levels were upregulated, along with a corresponding increase in pTrkB expression levels. This suggests that lycopene promotes BDNF secretion and restores the functional activity of the BDNF–TrkB pathway. Additionally, both PCR and WB analyses showed no significant changes in TrkB.FL levels among the three experimental groups, indicating that CSDS primarily affects the activity of the TrkB.FL receptor and downstream signaling pathways rather than directly altering receptor expression. Interestingly, we observed a downregulation of TrkB expression in the CSDS + Vehicle group. Studies have shown that TrkB is a truncated variant of TrkB.FL, containing only extracellular and transmembrane domains but lacking the intracellular kinase domain (Wang et al. 2024). While it shares a similar structure with TrkB.FL and can bind BDNF, it does not directly transmit BDNF–TrkB signaling. Instead, it functions as a regulator, competing for BDNF binding or forming heterodimers with TrkB.FL, thereby preventing further transmission of BDNF signals (Haapasalo et al. 2001). The trend of TrkB downregulation observed in the CSDS + Vehicle group aligns with the findings in study made by Wang et al. (2022), though they did not explore this in depth. We believe this may represent an adaptive mechanism attempting to enhance TrkB.FL signal transduction in depressive model mice. However, this adaptation is insufficient to fully restore normal signal transmission, which could explain the decreased pTrkB levels observed in the depressive model group.

In summary, our study reinforces the antidepressant effects of lycopene and further reveals that lycopene may exert its antidepressant effects by facilitating synaptic plasticity through the BDNF–TrkB pathway. These conclusions provide preclinical evidence for the potential clinical use of lycopene and introduce more possibilities for antidepressant treatment, such as serving as an adjunct therapy to SSRIs like fluoxetine. However, the efficacy of such combination therapy requires further investigation.

Undeniably, our research has its limitations. First, we only used male mice, which introduced a gender‐related limitation to our research. This choice was made because male mammals generally exhibit more robust hierarchical behavior and experience more significant stress than females in the context of CSDS (Atrooz, Alkadhi, and Salim 2021), which aligns better with our research focus. Second, we focused solely on the hippocampus as the region of interest. Other brain areas, like the prefrontal and cingulate cortex, are susceptible to stress as well (Chin Fatt et al. 2021). Future research will expand to include multiple brain regions. Finally, we only assessed changes in the BDNF–TrkB pathway without conducting reverse validation. For example, introducing BDNF or TrkB inhibitors followed by behavioral and protein changes in the mice could provide strong evidence for the involvement of this pathway. Additionally, considering that depression is a chronic disease, its pathological mechanisms often take longer to fully manifest. Therefore, extending the behavioral observation time after lycopene intervention may provide a more comprehensive evaluation of its efficacy, thus more accurately reflecting its effects and value in chronic disease intervention. We apologize for the limitations above and will strive to address them in future research.

## 5 Conclusions

For the first time, we have demonstrated the antidepressant effects of lycopene in a CSDS mouse model. Furthermore, we discovered that lycopene exerts its antidepressant effects by improving synaptic plasticity through the BDNF–TrkB pathway, which promotes the application of natural foods in depression treatment. In response to the limitations of this experiment, we plan to conduct further verification in future studies and include multiple brain regions in our research.

## Author Contributions

**Heyan Xu:** data curation (equal), validation (equal), writing – original draft (lead). **Yuna Wang:** investigation (equal), validation (equal). **Dandan Geng:** investigation (lead), validation (equal). **Fengming Chen:** conceptualization (equal), data curation (equal). **Yujia Chen:** data curation (equal), validation (equal). **Lisa Cynthia Niwenahisemo:** project administration (equal), validation (equal). **Lei Shi:** conceptualization (equal), methodology (equal). **Ning Du:** supervision (equal), validation (equal). **Ziqiang He:** software (equal), validation (equal), visualization (equal). **Xiaoming Xu:** project administration (equal), writing – review and editing (equal). **Li Kuang:** funding acquisition (equal), resources (equal), supervision (equal).

## Conflicts of Interest

The authors declare no conflicts of interest.

## Data Availability

The data that support the findings of this study are available from the corresponding author upon reasonable request.

## References

[fsn370003-bib-0001] Antoniuk, S. , M. Bijata , E. Ponimaskin , and J. Wlodarczyk . 2019. “Chronic Unpredictable Mild Stress for Modeling Depression in Rodents: Meta‐Analysis of Model Reliability.” Neuroscience & Biobehavioral Reviews 99: 101–116. 10.1016/j.neubiorev.2018.12.002. 30529362

[fsn370003-bib-0002] Atrooz, F. , K. A. Alkadhi , and S. Salim . 2021. “Understanding Stress: Insights From Rodent Models.” Current Research in Neurobiology 2: 100013. 10.1016/j.crneur.2021.100013. 36246514 PMC9559100

[fsn370003-bib-0003] Cao, T. , J. J. Matyas , C. L. Renn , A. I. Faden , S. G. Dorsey , and J. Wu . 2020. “Function and Mechanisms of Truncated BDNF Receptor TrkB.T1 in Neuropathic Pain.” Cells 9, no. 5: 1194. 10.3390/cells9051194. 32403409 PMC7290366

[fsn370003-bib-0004] Castrén, E. , and R. Hen . 2013. “Neuronal Plasticity and Antidepressant Actions.” Trends in Neurosciences 36, no. 5: 259–267. 10.1016/j.tins.2012.12.010. 23380665 PMC3648595

[fsn370003-bib-0005] Chen, D. , C. Huang , and Z. Chen . 2019. “A Review for the Pharmacological Effect of Lycopene in Central Nervous System Disorders.” Biomedicine & Pharmacotherapy 111: 791–801. 10.1016/j.biopha.2018.12.151. 30616078

[fsn370003-bib-0006] Chin Fatt, C. R. , C. Cooper , M. K. Jha , et al. 2021. “Dorsolateral Prefrontal Cortex and Subcallosal Cingulate Connectivity Show Preferential Antidepressant Response in Major Depressive Disorder.” Biological Psychiatry: Cognitive Neuroscience and Neuroimaging 6, no. 1: 20–28. 10.1016/j.bpsc.2020.06.019. 32921587 PMC10177661

[fsn370003-bib-0007] Cui, L. , S. Li , S. Wang , et al. 2024. “Major Depressive Disorder: Hypothesis, Mechanism, Prevention and Treatment.” Signal Transduction and Targeted Therapy 9, no. 1: 30. 10.1038/s41392-024-01738-y. 38331979 PMC10853571

[fsn370003-bib-0008] Dorsey, S. G. , R. M. Lovering , C. L. Renn , et al. 2012. “Genetic Deletion of trkB.T1 Increases Neuromuscular Function.” American Journal of Physiology. Cell Physiology 302, no. 1: C141–C153. 10.1152/ajpcell.00469.2010. 21865582 PMC3328911

[fsn370003-bib-0009] Duman, R. S. , G. K. Aghajanian , G. Sanacora , and J. H. Krystal . 2016. “Synaptic Plasticity and Depression: New Insights From Stress and Rapid‐Acting Antidepressants.” Nature Medicine 22, no. 3: 238–249. 10.1038/nm.4050. PMC5405628 26937618

[fsn370003-bib-0010] Fan, C. , Q. Song , P. Wang , Y. Li , M. Yang , and S. Y. Yu . 2019. “Neuroprotective Effects of Curcumin on IL‐1β‐Induced Neuronal Apoptosis and Depression‐Like Behaviors Caused by Chronic Stress in Rats.” Frontiers in Cellular Neuroscience 12: 516. 10.3389/fncel.2018.00516. 30666189 PMC6330766

[fsn370003-bib-0011] Ford, N. A. , A. C. Elsen , and J. W. Erdman . 2013. “Genetic Ablation of Carotene Oxygenases and Consumption of Lycopene or Tomato Powder Diets Modulate Carotenoid and Lipid Metabolism in Mice.” Nutrition Research 33, no. 9: 733–742. 10.1016/j.nutres.2013.07.007. 24034573 PMC3804893

[fsn370003-bib-0012] Fries, G. R. , V. A. Saldana , J. Finnstein , and T. Rein . 2023. “Molecular Pathways of Major Depressive Disorder Converge on the Synapse.” Molecular Psychiatry 28, no. 1: 284–297. 10.1038/s41380-022-01806-1. 36203007 PMC9540059

[fsn370003-bib-0013] Guo, Y. , Z. Fan , S. Zhao , et al. 2023. “Brain‐Targeted Lycopene‐Loaded Microemulsion Modulates Neuroinflammation, Oxidative Stress, Apoptosis and Synaptic Plasticity in β‐Amyloid‐Induced Alzheimer's Disease Mice.” Neurological Research 45, no. 8: 753–764. 10.1080/01616412.2023.2203615. 37068195

[fsn370003-bib-0014] Guo, Y. , X. Mao , J. Zhang , et al. 2019. “Oral Delivery of Lycopene‐Loaded Microemulsion for Brain‐Targeting: Preparation, Characterization, Pharmacokinetic Evaluation and Tissue Distribution.” Drug Delivery 26, no. 1: 1191–1205. 10.1080/10717544.2019.1689312. 31738085 PMC6882477

[fsn370003-bib-0015] Haapasalo, A. , E. Koponen , E. Hoppe , G. Wong , and E. Castren . 2001. “Truncated trkB.T1 Is Dominant Negative Inhibitor of trkB.TK1‐Mediated Cell Survival.” Biochemical and Biophysical Research Communications 280, no. 5: 1352–1358. 10.1006/bbrc.2001.4296. 11162678

[fsn370003-bib-0016] Horka, P. , V. Langova , J. Hubeny , K. Vales , I. Chrtkova , and J. Horacek . 2024. “Open Field Test for the Assessment of Anxiety‐Like Behavior in *Gnathonemus petersii* Fish.” Frontiers in Behavioral Neuroscience 17: 1280608. 10.3389/fnbeh.2023.1280608. 38268794 PMC10806096

[fsn370003-bib-0017] Howard, D. M. , M. J. Adams , T.‐K. Clarke , et al. 2019. “Genome‐Wide Meta‐Analysis of Depression Identifies 102 Independent Variants and Highlights the Importance of the Prefrontal Brain Regions.” Nature Neuroscience 22, no. 3: 343–352. 10.1038/s41593-018-0326-7. 30718901 PMC6522363

[fsn370003-bib-0018] Iñiguez, S. D. , A. Aubry , L. M. Riggs , et al. 2016. “Social Defeat Stress Induces Depression‐Like Behavior and Alters Spine Morphology in the Hippocampus of Adolescent Male C57BL/6 Mice.” Neurobiology of Stress 5: 54–64. 10.1016/j.ynstr.2016.07.001. 27981196 PMC5154707

[fsn370003-bib-0019] James, S. L. , D. Abate , K. H. Abate , et al. 2018. “Global, Regional, and National Incidence, Prevalence, and Years Lived With Disability for 354 Diseases and Injuries for 195 Countries and Territories, 1990–2017: A Systematic Analysis for the Global Burden of Disease Study 2017.” Lancet 392, no. 10159: 1789–1858. 10.1016/S0140-6736(18)32279-7. 30496104 PMC6227754

[fsn370003-bib-0020] Khalifeh, M. , R. Hobeika , L. El Hayek , et al. 2020. “Nicotine Induces Resilience to Chronic Social Defeat Stress in a Mouse Model of Water Pipe Tobacco Exposure by Activating BDNF Signaling.” Behavioural Brain Research 382: 112499. 10.1016/j.bbr.2020.112499. 31978493

[fsn370003-bib-0021] Kumar, P. V. N. , P. Elango , S. Asmathulla , and S. Kavimani . 2019. “Lycopene Treatment Transposed Antidepressant‐Like Action in Rats Provoked to Chronic Mild Stress.” Biomedical and Pharmacology Journal 12, no. 2: 981–988. 10.13005/bpj/1725.

[fsn370003-bib-0022] Lim, B. K. , K. W. Huang , B. A. Grueter , P. E. Rothwell , and R. C. Malenka . 2012. “Anhedonia Requires MC4R‐Mediated Synaptic Adaptations in Nucleus Accumbens.” Nature 487, no. 7406: 183–189. 10.1038/nature11160. 22785313 PMC3397405

[fsn370003-bib-0023] Lin, H.‐Y. , B.‐R. Huang , W.‐L. Yeh , et al. 2014. “Antineuroinflammatory Effects of Lycopene via Activation of Adenosine Monophosphate‐Activated Protein Kinase‐α1/Heme Oxygenase‐1 Pathways.” Neurobiology of Aging 35, no. 1: 191–202. 10.1016/j.neurobiolaging.2013.06.020. 23906616

[fsn370003-bib-0024] Lin, J. , J. Xia , H.‐S. Zhao , et al. 2018. “Lycopene Triggers Nrf2–AMPK Cross Talk to Alleviate Atrazine‐Induced Nephrotoxicity in Mice.” Journal of Agricultural and Food Chemistry 66, no. 46: 12385–12394. 10.1021/acs.jafc.8b04341. 30360616

[fsn370003-bib-0025] Liu, M.‐Y. , C.‐Y. Yin , L.‐J. Zhu , et al. 2018. “Sucrose Preference Test for Measurement of Stress‐Induced Anhedonia in Mice.” Nature Protocols 13, no. 7: 1686–1698. 10.1038/s41596-018-0011-z. 29988104

[fsn370003-bib-0026] Lu, J. , X. Gong , X. Yao , et al. 2021. “Prolonged Chronic Social Defeat Stress Promotes Less Resilience and Higher Uniformity in Depression‐Like Behaviors in Adult Male Mice.” Biochemical and Biophysical Research Communications 553: 107–113. 10.1016/j.bbrc.2021.03.058. 33765554

[fsn370003-bib-0027] Malhi, G. S. , and J. J. Mann . 2018. “Depression.” Lancet 392, no. 10161: 2299–2312. 10.1016/S0140-6736(18)31948-2. 30396512

[fsn370003-bib-0028] McEwen, B. , C. Nasca , and J. Gray . 2016. “Stress Effects on Neuronal Structure: Hippocampus, Amygdala, and Prefrontal Cortex.” Neuropsychopharmacology 41: 3–23. 10.1038/npp.2015.171. 26076834 PMC4677120

[fsn370003-bib-0029] Moda‐Sava, R. N. , M. H. Murdock , P. K. Parekh , et al. 2019. “Sustained Rescue of Prefrontal Circuit Dysfunction by Antidepressant‐Induced Spine Formation.” Science 364, no. 6436: eaat8078. 10.1126/science.aat8078. 30975859 PMC6785189

[fsn370003-bib-0030] Nagy, C. , M. Maitra , A. Tanti , et al. 2020. “Single‐Nucleus Transcriptomics of the Prefrontal Cortex in Major Depressive Disorder Implicates Oligodendrocyte Precursor Cells and Excitatory Neurons.” Nature Neuroscience 23, no. 6: 771–781. 10.1038/s41593-020-0621-y. 32341540

[fsn370003-bib-0031] Podyma, B. , K. Parekh , A. D. Güler , and C. D. Deppmann . 2021. “Metabolic Homeostasis via BDNF and Its Receptors.” Trends in Endocrinology and Metabolism 32, no. 7: 488–499. 10.1016/j.tem.2021.04.005. 33958275 PMC8192464

[fsn370003-bib-0032] Renn, C. L. , C. C. Leitch , and S. G. Dorsey . 2009. “In Vivo Evidence That Truncated Trkb.T1 Participates in Nociception.” Molecular Pain 5: 61. 10.1186/1744-8069-5-61. 19874592 PMC2777863

[fsn370003-bib-0033] Ru, Q. , Y. Lu , A. B. Saifullah , et al. 2022. “TIAM1‐Mediated Synaptic Plasticity Underlies Comorbid Depression–Like and Ketamine Antidepressant–Like Actions in Chronic Pain.” Journal of Clinical Investigation 132, no. 24: e158545. 10.1172/JCI158545. 36519542 PMC9753999

[fsn370003-bib-0034] Saini, R. K. , K. R. R. Rengasamy , F. M. Mahomoodally , and Y.‐S. Keum . 2020. “Protective Effects of Lycopene in Cancer, Cardiovascular, and Neurodegenerative Diseases: An Update on Epidemiological and Mechanistic Perspectives.” Pharmacological Research 155: 104730. 10.1016/j.phrs.2020.104730. 32126272

[fsn370003-bib-0035] Sairanen, M. , G. Lucas , P. Ernfors , M. Castrén , and E. Castrén . 2005. “Brain‐Derived Neurotrophic Factor and Antidepressant Drugs Have Different but Coordinated Effects on Neuronal Turnover, Proliferation, and Survival in the Adult Dentate Gyrus.” Journal of Neuroscience 25, no. 5: 1089–1094. 10.1523/JNEUROSCI.3741-04.2005. 15689544 PMC6725966

[fsn370003-bib-0036] Samarghandian, S. , M. Azimi‐Nezhad , T. Farkhondeh , and F. Samini . 2017. “Anti‐Oxidative Effects of Curcumin on Immobilization‐Induced Oxidative Stress in Rat Brain, Liver and Kidney.” Biomedicine & Pharmacotherapy 87: 223–229. 10.1016/j.biopha.2016.12.105. 28061405

[fsn370003-bib-0037] Slavich, G. M. , and J. Sacher . 2019. “Stress, Sex Hormones, Inflammation, and Major Depressive Disorder: Extending Social Signal Transduction Theory of Depression to Account for Sex Differences in Mood Disorders.” Psychopharmacology 236, no. 10: 3063–3079. 10.1007/s00213-019-05326-9. 31359117 PMC6821593

[fsn370003-bib-0038] Trumbo, P. R. 2005. “Are There Adverse Effects of Lycopene Exposure?” Journal of Nutrition 135, no. 8: 2060S–2061S. 10.1093/jn/135.8.2060S. 16046742

[fsn370003-bib-0039] U.S. Food and Drug Administration . 2002. “Guidance for Industry and Reviewers: Estimating the Safe Starting Dose in Clinical Trials for Therapeutics in Adult Healthy Volunteers.” FDA. https://www.fda.gov.

[fsn370003-bib-0040] Wang, C. , E. Kavalali , and L. Monteggia . 2023. “BDNF Signaling in Context: From Synaptic Regulation to Psychiatric Disorders.” Cell 185, no. 1: 62–76. 10.1016/j.cell.2021.12.003. PMC8741740 34963057

[fsn370003-bib-0041] Wang, J. , H.‐S. Chen , H.‐H. Li , et al. 2023. “Microglia‐Dependent Excessive Synaptic Pruning Leads to Cortical Underconnectivity and Behavioral Abnormality Following Chronic Social Defeat Stress in Mice.” Brain, Behavior, and Immunity 109: 23–36. 10.1016/j.bbi.2022.12.019. 36581303

[fsn370003-bib-0042] Wang, Y. , J. Liang , B. Xu , J. Yang , Z. Wu , and L. Cheng . 2024. “TrkB/BDNF Signaling Pathway and Its Small Molecular Agonists in CNS Injury.” Life Sciences 336: 122282. 10.1016/j.lfs.2023.122282. 38008209

[fsn370003-bib-0043] Wang, Y.‐L. , C.‐C. Chio , S.‐C. Kuo , et al. 2022. “Exercise Rehabilitation and/or Astragaloside Attenuate Amyloid‐Beta Pathology by Reversing BDNF/TrkB Signaling Deficits and Mitochondrial Dysfunction.” Molecular Neurobiology 59, no. 5: 3091–3109. 10.1007/s12035-022-02728-3. 35262870

[fsn370003-bib-0044] Warden, D. , A. J. Rush , M. H. Trivedi , M. Fava , and S. R. Wisniewski . 2007. “The STAR*D Project Results: A Comprehensive Review of Findings.” Current Psychiatry Reports 9, no. 6: 449–459. 10.1007/s11920-007-0061-3. 18221624

[fsn370003-bib-0045] Yan, Y. , X. Xu , R. Chen , et al. 2021. “Down‐Regulation of MST1 in Hippocampus Protects Against Stress‐Induced Depression‐Like Behaviours and Synaptic Plasticity Impairments.” Brain, Behavior, and Immunity 94: 196–209. 10.1016/j.bbi.2021.02.007. 33607238

[fsn370003-bib-0046] Yu, Q. , F. Xue , Z. Li , et al. 2022. “Dietary Intake of Carotenoids and Risk of Depressive Symptoms: A Systematic Review and Meta‐Analysis.” Antioxidants 11, no. 11: 2205. 10.3390/antiox11112205. 36358577 PMC9686905

[fsn370003-bib-0047] Yuen, E. Y. , J. Wei , W. Liu , P. Zhong , X. Li , and Z. Yan . 2012. “Repeated Stress Causes Cognitive Impairment by Suppressing Glutamate Receptor Expression and Function in Prefrontal Cortex.” Neuron 73, no. 5: 962–977. 10.1016/j.neuron.2011.12.033. 22405206 PMC3302010

[fsn370003-bib-0048] Zhang, F. , Y. Fu , X. Zhou , et al. 2016. “Depression‐Like Behaviors and Heme Oxygenase‐1 Are Regulated by Lycopene in Lipopolysaccharide‐Induced Neuroinflammation.” Journal of Neuroimmunology 298: 1–8. 10.1016/j.jneuroim.2016.06.001. 27609268

[fsn370003-bib-0049] Zhao, B. , H. Liu , J. Wang , et al. 2018. “Lycopene Supplementation Attenuates Oxidative Stress, Neuroinflammation, and Cognitive Impairment in Aged CD‐1 Mice.” Journal of Agricultural and Food Chemistry 66, no. 12: 3127–3136. 10.1021/acs.jafc.7b05770. 29509007

